# Genomic Imprinting, Epigenetic Dysregulation, and Neuropsychiatric Mechanisms in Prader–Willi Syndrome: A Multi-Level Integrative Review

**DOI:** 10.3390/cells15030268

**Published:** 2026-01-31

**Authors:** Zofia Śledzikowska, Xawery Eryk Żukow, Zuzanna Małgorzata Antos, Napoleon Waszkiewicz

**Affiliations:** 1 Faculty of Medicine, Medical University of Białystok, Jana Kilińskiego 1, 15-089 Białystok, Poland; 39714@student.umb.edu.pl (X.E.Ż.); 39615@student.umb.edu.pl (Z.M.A.); 2 Department of Psychiatry, Medical University of Bialystok, Michała Wołodyjowskiego 2, 15-272 Białystok, Poland; napoleon.waszkiewicz@umb.edu.pl

**Keywords:** genomic imprinting, Prader–Willi syndrome, epigenetic dysregulation, *SNORD116*, *MAGEL2*, gene regulatory networks, chromatin architecture, non-coding RNAs

## Abstract

**Highlights:**

**What are the main findings?**

- Genomic imprinting defects at the chromosome region 15q11-q13 are responsible for epigenetic and transcriptional abnormalities that contribute to neurodevelopmental vulnerability in individuals with Prader–Willi syndrome.
- Deletion-type and maternal uniparental disomy (mUPD) forms of Prader–Willi syndrome involve distinct molecular pathways that partially overlap in their downstream effects on neurotransmitter systems and neural circuitry.

**What is the implication of the main finding?**

- Prader–Willi syndrome is an example of a natural model that relates psychiatric phenotype to the regulation of genes by imprinting-dependent mechanisms.
- Understanding shared and subtype-specific pathways may inform biomarker development and mechanism-based stratification in neuropsychiatric research.

**Abstract:**

Prader–Willi syndrome (PWS) is a rare imprinting-related neurodevelopmental disorder caused by loss of paternally expressed genes within the chromosome 15q11–q13 region, including SNORD116, MAGEL2, and NDN. It provides a natural model for examining how genomic imprinting disruptions shape neural development and psychiatric vulnerability. This review synthesizes current evidence to clarify the mechanistic pathways linking imprinting defects and epigenetic dysregulation to neuropsychiatric outcomes in PWS. Published studies—including patient-derived induced pluripotent stem cell (iPSC) models, animal knockout systems (e.g., Magel2-null models), transcriptomic and DNA methylation datasets, and human neuroimaging research—were identified through targeted searches of PubMed and Web of Science and integrated narratively rather than through systematic procedures. Across these data sources, deletion-type PWS is primarily associated with impaired neuronal maturation, altered serotonergic signaling, and locus-specific transcriptional dysregulation. Maternal uniparental disomy (mUPD) is characterized by broader epigenetic alterations within the imprinted domain, genome-wide transcriptional effects, dopaminergic pathway alterations, and disrupted prefrontal–limbic connectivity linked to increased psychosis risk. Importantly, available evidence supports substantial phenotypic and mechanistic overlap between PWS subtypes, with genotype–phenotype associations reflecting probabilistic tendencies rather than categorical distinctions. Collectively, convergent findings across molecular, neurochemical, and systems-level studies support a mechanistic continuum extending from imprinting defects to behavioral phenotypes. These insights position PWS as a translational model for understanding how epigenetic dysregulation contributes to psychiatric risk and highlight the need for genotype-informed, mechanistically grounded research to advance biomarker development and targeted therapeutic strategies.

## 1. Introduction

Prader–Willi Syndrome (PWS) is a rare neurodevelopmental disorder caused by the lack of expression of paternal imprinted genes located on chromosome 15q11–q13 [1,2]. The syndrome results from several distinct genetic mechanisms, including paternal deletions, maternal uniparental disomy (mUPD), or imprinting center defects [3]. All of them lead to the functional absence of paternally expressed genes critical for brain development and neurobehavioral regulation [1,4]. Among the most well-studied genes within the PWS locus are *SNORD116* (small nucleolar RNA, C/D box 116), *MAGEL2* (melanoma antigen-like 2), and *NDN* (*necdin*), which have been shown to be important for brain development, synaptic function, and circadian regulation [5,6]. *SNORD116* is a cluster of paternally expressed non-coding RNA important for normal hypothalamic development and neuronal maturation. The loss of these genes has been identified as a primary molecular cause of the PWS phenotype [7]. *MAGEL2* encodes a ubiquitin pathway-associated protein that regulates circadian rhythm, synaptic activity, and neuroendocrine signaling. Additionally, *MAGEL2* variants are implicated in the Schaaf–Yang syndrome [7,8,9]. *NDN* has been found to play a role in neuronal differentiation, axonal elongation, and survival, emphasizing the importance of this imprinted region in neurodevelopment [7].

The estimated global prevalence of PWS is between 1 in 10,000 and 1 in 30,000 live births, and it is consistently observed across populations [1]. Although significant advancements have been made in the field of genetic diagnosis and early detection of PWS, it remains a lifelong condition with complex metabolic, cognitive, and psychiatric manifestations [1,10]. Current clinical management of PWS is primarily supportive and multidisciplinary, focusing on growth hormone replacement, nutritional regulation, behavioral interventions, and treatment of psychiatric comorbidities [11,12,13,14]. As of today, there are no disease-modifying or mechanism-based treatments for PWS; thus, further mechanistic understanding of the relationship between imprinting defects and neurodevelopmental and neuropsychiatric outcomes is necessary [13,15,16]. In this context, PWS represents a unique human model for investigating how epigenetic dysregulation of imprinted genes disrupts molecular, neurochemical, and circuit-level processes in the brain. This review integrates evidence from cellular models, animal studies, transcriptomic and epigenetic analysis, and human imaging studies to explain pathways linking genomic imprinting defects to behavioral and psychiatric phenotypes in PWS.

Historically characterized by metabolic and endocrine symptoms, PWS also offers a powerful natural experiment to explore how imprinting-sensitive molecular pathways regulate neuronal development and create vulnerability to psychiatric illness. Beyond hypotonia, hyperphagia, and cognitive impairment, individuals with PWS often exhibit affective instability, compulsivity, and—in specific genotypes—enhanced risk of psychosis-like features, collectively indicating significant neuropsychiatric burden.

This review provides a mechanistic, neurobiological, and translational overview of how genomic imprinting defects lead to neurobehavioral phenotypes in PWS. The two main molecular causes of PWS, paternal deletion and maternal uniparental disomy (mUPD), account for >95% of cases [1]. Both subtypes have distinct downstream neurobiological consequences. Individuals with deletion-type PWS have exhibited greater levels of emotional dysregulation, compulsive behaviors, and cognitive inflexibility than those with mUPD. Patients with mUPD have a higher incidence of mood disturbances and psychosis-like presentations compared to those with deletion-type PWS in certain cohorts. However, the same symptomatology is present in individuals with both deletion-type and mUPD PWS. This convergence suggests that shared clinical features may reflect common downstream effects of disrupted imprinting on neurodevelopmental and neuromodulatory systems, rather than subtype-specific psychopathology.

Emerging mechanistic and preclinical data now uncover how loss of paternally expressed imprinted genes such as *SNORD116*, *MAGEL2*, and *NECDIN* (*NDN*) disrupts neuronal maturation, synaptic plasticity, and neuromodulatory regulation—particularly within serotonergic, dopaminergic, and hypothalamic networks [15,16,17]. In turn, these molecular and cellular impairments can produce abnormal circuitry connections, neurotransmitter imbalance, and a predisposition to neurobehavioral dysfunction. Animal models lacking either *SNORD116* or *MAGEL2* mimic many characteristics of the PWS phenotype, showing that dysregulation of non-coding RNA and imprinted proteins dependent on imprinting can significantly affect the brain and its development [7,15].

Although there is an expanding body of biological knowledge regarding the psychiatric issues associated with Prader–Willi Syndrome (PWS), the psychiatric component of PWS is still grossly under-characterized. Most of the scientific research to date has been directed at understanding the somatic, metabolic, and developmental dimensions of PWS. Few working models have integrated the effects of genomic dysfunction, imbalanced neurotransmitter levels, and the pathological effects of dysfunctional neural circuits.

To address current gaps in the literature, this review synthesizes evidence found in the literature that relates to molecular, neuromodulatory, and system-wide dimensions of PWS. It focuses specifically on the ways in which defects in genomic imprinting can influence neurodevelopmental trajectories and the psychiatric risks associated with PWS. The review also examines the potential similarities and differences in the types of behavioral and cognitive vulnerabilities exhibited by individuals with either deletions or uniparental disomy (mUPD) of chromosome 15q11-q13.

In addition to comparing potential differences in the types of behavioral and cognitive vulnerabilities associated with deletions versus mUPDs of chromosome 15q11-q13, this review discusses the implications for identifying vulnerable populations. It may provide guidance on developing cautious, precision-based interventions for those at risk of exhibiting significant behavioral and cognitive problems due to the presence of deletion or mUPD of chromosome 15q11-q13. These discussions are intended to reflect tendencies arising from overlapping neurodevelopmental and neuromodulatory mechanisms and should be viewed as representing non-discrete or non-overlapping phenotypic categories.

In light of the increasing interest in the role of imprinting-regulated non-coding RNA, chromatin organization, and the regulation of neuronal differentiation, Prader–Willi Syndrome (PWS) provides a unique framework for studying the molecular/cellular basis of PWS, allowing for a mechanistic focus in cells.

This review provides a particularly detailed examination of the molecular, epigenetic, and cellular pathways that link defects in genomic imprinting to the development of abnormal neurons and the subsequent neurobehavioral phenotypes associated with PWS.

This article represents a narrative, mechanistic review of the available literature that integrates genetic, epigenetic, molecular neuroscientific, neurochemical, and neuroimaging studies of PWS. The literature reviewed herein was identified using targeted searches of PubMed, Scopus, and Web of Science (articles published from 2000 to 2025) using combinations of search terms including Prader–Willi Syndrome, genomic imprinting, *SNORD116*, *MAGEL2*, psychiatric phenotypes, epigenetics, dopamine, and serotonin. Additionally, relevant studies were identified using citation tracking and area-specific expertise.

Peer-reviewed original research articles, mechanistic experimental studies, large cohort investigations, and high-quality neuroimaging studies that examined genotype-dependent neurobiological mechanisms were given priority in this review. Due to the focus of this review being on conceptual and mechanistic integration rather than on systematic evidence synthesis, formal PRISMA methodology was not employed in this study.

Studies that were peer-reviewed original research articles, mechanistic experimental studies, large cohort studies, and high-quality neuroimaging studies that examined genotype-dependent molecular, cellular, or circuit-level mechanisms in PWS were included in the review. Both human studies and relevant animal or patient-derived cellular models were included in the review when they provided mechanistic insights into imprinting-related neurodevelopmental processes. The studies that were excluded from the review were those that were (1) limited to isolated case reports that lacked direct relevance to mechanistic explanations of imprinting-related neurodevelopmental processes, (2) limited to descriptive clinical reports of PWS that lacked neurobiological interpretations, and (3) limited to articles that did not directly relate to PWS or to pathways dependent upon genomic imprinting.

## 2. Mechanistic Basis of PWS

Psychiatric variability seen in Prader–Willi Syndrome (PWS) can be traced back to the genomic and epigenetic factors that cause it. The majority (>70%) of PWS are caused by a paternal deletion of the 15q11-q13 region, and the remainder of individuals with PWS have maternal uniparental disomy (mUPD) of chromosome 15 (~20–30%). As these two mechanisms result in the loss of expression of paternally expressed imprinted genes, they generate different molecular effects as a result—neurodevelopmental pathways/neurobehavioral profiles which are partially separable yet highly interrelated [1,2].

**Figure 1:**
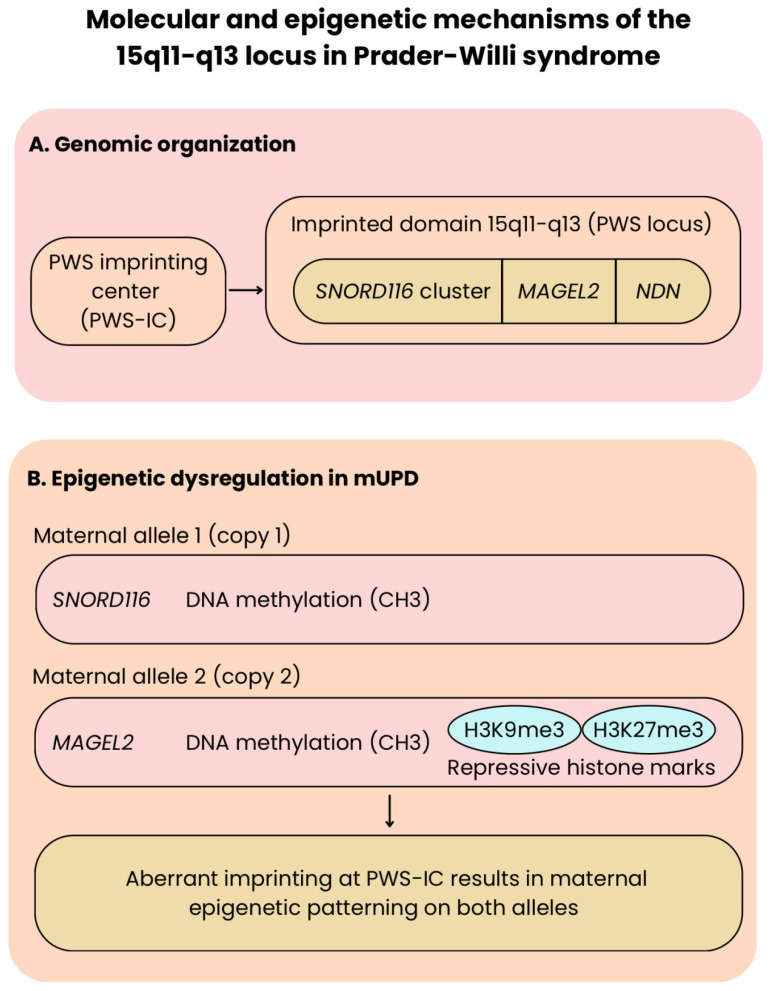
Molecular and epigenetic mechanisms of the 15q11–q13 locus in Prader–Willi syndrome. (**A**) Genomic organization of the imprinted 15q11–q13 region (PWS locus), highlighting the relative positions of the PWS imprinting center (PWS-IC) and key paternally expressed genes, including the *SNORD116* cluster, *MAGEL2*, and *NDN*. Gene positions are shown schematically and are not drawn to the genomic scale. (**B**) Panel B illustrates epigenetic mechanisms that have been experimentally documented at specific loci within the PWS region. DNA methylation at the PWS imprinting center is sufficient to silence the SNORD116 cluster, whereas repression of MAGEL2 has been shown to involve both DNA methylation and repressive histone modifications (H3K9me3 and H3K27me3).

Figure 1 shows the arrangement of the 15q11-q13 region of the genome and the parental origin-specific expression of several key imprinted genes.

## 3. Deletion Mechanisms

Paternal deletions delete several key imprinted elements, including the noncoding *SNORD116* snoRNA cluster and the protein-coding imprinted genes *MAGEL2*, *NECDIN* (*NDN*), *SNURF-SNRPN*, and many others that regulate imprinted domains [2,15]. The *SNORD116* cluster has been identified as the molecular hub through which the loss of imprinting causes neuronal dysregulation [10,16]. It is believed that the noncoding RNA from this cluster plays a role in regulating posttranscriptional gene expression, RNA stability, alternative splicing, and chromatin remodeling in neuronal precursor cells, which are necessary for appropriate neuronal development and synaptic maturation [17,18,19]. Recent high-resolution analyses further demonstrated that loss of *SNORD116* disrupts neuron-specific alternative splicing mediated by Rbfox3/NeuN, alters 3D chromatin interactions within the PWS imprinting domain, and misregulates gene networks involved in neurogenesis and synaptic assembly. Together, these findings indicate that *SNORD116* may play a crucial role in the posttranscriptional regulation of neuronal cell fate and the link between the loss of imprinting and the vulnerability of neurons [17,20].

Mouse model studies that were genetically engineered to be missing *SNORD116*, *MAGEL2*, or *NDN* have shown that the loss of these elements disrupts the process of differentiating into neurons, the formation of synapses, and the regulation of neurotransmitters [15,17]. For example, mice that lack *MAGEL2* exhibit disruptions in the secretion of hypothalamic neuropeptides, disruptions in circadian regulation, impairments in synaptic receptor trafficking (e.g., AMPA receptor cycling), and an imbalance in monoaminergic systems (serotonin and dopamine), which can cause disruptions in the regulation of emotions, reward processing, and behavioral control [7,21]. Similar results have also been found in mice that are deficient in *NDN*, but the deficiencies are limited to disruptions in the final stages of neuronal differentiation, axon growth, and the ability of neurons to withstand metabolic or oxidative stress. Collectively, these alterations may compromise the inhibitory-excitatory balance in the limbic system, which is important for regulating affect and cognitive flexibility [2,17].

Therefore, deletions of paternal DNA are associated with a series of disruptions at the molecular level that begin with the loss of genomic imprinting and the loss of expression of noncoding RNAs and proteins. The primary consequences of imprinting loss are thought to involve dysregulation of gene regulatory networks, leading to impaired synapse development and altered neurotransmitter signaling. These molecular and synaptic alterations may subsequently affect neural circuit function, providing a mechanistic basis for the higher prevalence of affective dysregulation, compulsive behaviors, and cognitive inflexibility observed in individuals with deletion-type PWS compared with those with maternal uniparental disomy.

## 4. Epigenetic Dysregulation in mUPD

Maternal uniparental disomy (mUPD) causes both chromosomes 15 to be inherited from the mother, thereby completely silencing all of the paternally expressed imprinted loci—including *SNORD116*, *MAGEL2*, *NDN*, etc. They are subject to parent-of-origin-specific epigenetic silencing [1,10] by virtue of methylation of the imprinting center (PWS-IC), along with histone modifications, that keep the maternal allele locked into an active transcriptional state. More recent mechanistic studies have shown that several chromatin regulators—for example, histone methyltransferase EHMT2—play a role in maintaining the silenced state of maternal imprinted alleles by placing repressive histone marks (for example, H3K9me2) and causing 3D-chromatin compaction [5,22]. Thus, mUPD removes gene products but can also alter the epigenetic landscape and transcriptional regulation in neural cells across the genome, potentially affecting many downstream pathways of the classical PWS locus.

At the chromatin level, the silenced state produced by mUPD is maintained by a multilayered process involving the coordinated placement of repressive histone marks such as H3K9me2/3 and dense DNA methylation at the imprinting control region. The combination of these two types of epigenetic modification creates a compacted chromatin structure that is resistant to transcriptional activation. In addition to maintaining the silenced state of paternal alleles, this multi-layered repression has a secondary effect of creating additional transcriptional instability in synaptic, metabolic, and neurodevelopmental gene networks and increasing the cellular vulnerability to environmental insults.

Studies using neuronal cultures created from patient-derived iPSCs and from animal models indicate that the loss of imprinting leads to disruption of expression of multiple genes related to neurodevelopment, synaptic function, neurotransmitter regulation, and circadian biology—many of which are located outside the 15q11-q13 region itself [17,23,24]. The broad disruptions in transcription may lead to instability within neurons, abnormal connectivity between them, and imbalances in their modulatory signals, which could contribute to the increased risks of mood disorder, psychosis-like behaviors, and affective instability commonly seen in mUPD patients.

Finally, therapeutic and experimental approaches to reactivate silenced paternal alleles (for example, locus-specific epigenome editing) are being tested and developed, suggesting that PWS may not only serve as a disease model for studying imprinting disorders but also as an opportunity to develop epigenetic therapies for treating such disorders [22,25].

The epigenetic effects of mUPD at the PWS imprinting center are shown schematically in Figure 1.

## 5. Comparative Insights: Imprinting Defects as a Model for Behavioral–Cognitive Vulnerability

A comparison of the different trajectories of neuropsychiatric and behavioral development among individuals with deletion-type and mUPD genotypes illustrates the impact of parental origin on the expression of differing patterns of development. Deletion carriers show an overwhelming majority of early onset emotion dysregulation and compulsive behaviors based on synaptic and neuromodulatory impairment. In contrast, mUPD carriers have shown the most common occurrence of late-developing mood disorder, psychosis like symptoms, and network-level dysfunction likely due to the greater breadth of epigenetic disruption and network destabilization.

These two distinct genotypes create a mechanism for examining the degree to which imprinting-sensitive regulation of non-coding RNA and chromatin machinery contribute to neurodevelopment, neurotransmission, and the likelihood of developing certain types of behavioral/cognitive vulnerabilities. Additionally, the study of PWS could yield generalizable knowledge for other imprinting disorders (such as Angelman Syndrome) or idiopathic psychiatric disorders that result in epigenetic disruption, providing translational relevance for the study of rare diseases to the study of more common disorders.

Key mechanistic differences between deletion-type PWS and mUPD-PWS are illustrated in Table 1.

Also, emerging data suggest that there is a significant probability that some degree of overlap exists regarding the behavioral, cognitive, and psychiatric manifestations among deletion type and mUPD Prader–Willi syndrome. The degree of overlap reflects the convergence of the final common pathways of neurodevelopmental and neuromodulatory processes, which are influenced by developmental timing, the epigenetic context, and environmental factors.

## 6. Neurobiological Mechanisms

The emergence of neurobehavioral characteristics of Prader–Willi Syndrome (PWS) occurs due to disruptions at many different biological levels—including genomic imprinting, molecular/epigenetic disruption, neurochemical imbalance, and neural network disruption. The loss of paternal expression of imprinted genes such as *SNORD116*, *MAGEL2*, and *NECDIN*/*NDN* results in the abnormal regulation of cells, which disrupts normal neuronal development and function of both neuromodulatory systems and the overall structure of networks [1,10,15]. Both *MAGEL2* and *NECDIN* are involved in the ubiquitin-proteasome pathway of protein degradation and the endosomal recycling of proteins, and therefore, the disruption of imprinted genes could disrupt the trafficking of AMPAR/NMDAR receptors to the synapse and the intracellular signaling cascades important for neuronal maturation. Furthermore, abnormalities in the structural organization of the cytoskeleton and axonal transport have been demonstrated in neurons lacking *Magel2* that indicate cell autonomous mechanism(s) by which circuits can become vulnerable.

### 6.1. Molecular and Epigenetic Disruption: From Imprinting to Neuronal Dysfunction

More than just protein-coding mRNAs are affected when deletion or inactivation of paternally expressed imprinted genes occurs. Small nucleolar RNAs (snoRNAs), encoded in the *SNORD116* cluster, are non-coding RNAs that likely modulate either post-transcriptional events, RNA stability, or chromatin structure in neurons [16,20]. Loss of *SNORD116* expression in neuronal cell culture models changes the expression of numerous genes predicted to be regulated by *SNORD116*: in human neurons from PWS models, dozens of genes involved in synaptic function and neurodevelopment show differential expression [17].

Additionally, protein-coding imprinted genes in the 15q11–q13 region of chromosome 15, like *MAGEL2* and *NDN*, are crucial for neuronal development, axon maintenance, synaptic receptor trafficking, and neuropeptide regulation [15,32]. Mouse knockout models for *Magel2* and *Ndn* mimic some aspects of the PWS phenotype, including disruption of hypothalamic neuropeptide secretion, abnormal circadian rhythms, disrupted sleep patterns, metabolic dysfunction, and behavioral abnormalities [7,15].

The functional implications of imprinted gene expression loss appear to result in impaired synaptic plasticity, receptor sorting (for example, impaired glutamate receptor trafficking), and formation of neural networks with increased risk of failure in corticolimbic circuitry (primarily demonstrated through molecular/cellular studies). Collectively, these data suggest that a mechanistic cascade is present where loss of genomic imprinting results in epigenetic/transcriptional dysregulation and subsequently impairs neurodevelopment/synaptic function, contributing to an imbalance in brain function due to altered neuromodulation.

These findings are preliminary, based mostly on uncontrolled clinical reports, and cannot be used as therapeutic guidance.

#### Insights from Patient-Derived iPSC Models

Patient-derived induced pluripotent stem cell (iPSC) data increasingly support a mechanistic link between an individual’s imprinting disorders and increased neuronal susceptibility in Prader–Willi Syndrome (PWS). In addition to exhibiting broad-scale transcriptional dysfunction in genes related to synaptic formation, neuronal maturation, and neurotransmission, iPSC-differentiated neurons isolated from individuals with PWS also showed transcriptional aberrations outside of the traditional 15q11–q13 locus that encompassed the original PWS locus. The widespread transcriptional dysregulation observed in iPSC-differentiated neurons from individuals with PWS is indicative of secondary downstream effects of imprinting loss on large-scale gene-regulatory networks [17,28]. Recent studies have shown that loss of *SNORD116* RNA expression in human neuronal cultures disrupts neuron-specific alternative splicing programs controlled by Rbfox3/NeuN, altered chromatin organization, and misregulated synaptic gene expression [17,20]. Together, findings from recent studies suggest that imprinting-dependent non-coding RNAs produce cell-autonomous influences on the maturation and/or identification of neurons [17,20]. In a similar fashion, iPSC-differentiated neurons lacking *MAGEL2* expression have impaired endosomal trafficking, synaptic receptor recycling, and circadian gene expression, indicating that ubiquitin–proteasome-related pathways are important for maintaining synaptic stability and regulating neuromodulatory activity [28]. Overall, the use of iPSC-based models provides a translationally relevant bridge between the molecular defects caused by imprinting disorders in humans and the resulting human neuronal phenotypes; therefore, they offer additional mechanistic insights into human disease that can be used to identify possible therapeutic targets for epigenetic or gene-reactivation therapies.

### 6.2. Neurochemical Imbalance and Circuit-Level Consequences

These molecular abnormalities lead to a high frequency of monoaminergic imbalance. Research using imprinted gene knockout mice has demonstrated alterations in serotonergic and dopaminergic neurotransmission, as well as disruptions in neuropeptide regulation and impaired hypothalamus and limbic system signaling [7,15].

Research based on available molecular and preclinical data suggest that deletions in Prader–Willi syndrome (PWS) due to predominant loss of *SNORD116*/*MAGEL2*/*NDN* are associated with compulsive behavior and emotional inflexibility as well as affective dysregulation that is consistent with the serotonergic hypoactivity seen in the *Magel2*-null model [20,27] and with the broader imprinting-related neuropsychiatric frameworks [33].

In contrast, in cases of maternal uniparental disomy (mUPD), silencing of the imprinting region is thought to cause broader disruption, affecting multiple other genes and epigenetic regulators [26]. These pathways have also been shown to affect dopaminergic signaling and reward processing [34] and to interact with broader changes in endocrine and neurodevelopmental function [35,36], similar to those reported by clinical studies examining monoaminergic balances in patients with mUPD [26,35].

Studies at the network level demonstrate that neuroimaging and connectomics support the notion of network-level abnormalities in PWS: structural and functional connectivity within the prefrontal-limbic, default-mode, and salience networks appears to be compromised, as evidenced by reduced white-matter integrity and altered functional coupling (note: detailed studies need careful matching; however, the convergence with molecular and neurochemical data is significant) [30,35].

Specifically, resting-state fMRI studies have found reduced hypothalamic-limbic functional connectivity [35] and disrupted intra- and inter-network coupling within the default mode and salience systems [30], which supports the idea that the consequences of imprinting defects extend to large-scale neural networks.

Additionally, peripheral metabolic and hormonal disturbances characteristic of PWS (such as hypothalamic dysfunction, energy imbalance, and neuropeptide dysregulation) have been proposed to increase the vulnerability of these networks to further damage through neuroinflammation [37,38], impaired neurotrophic support, and altered neuromodulator synthesis—mechanisms increasingly recognized in the pathophysiology of neuropsychiatry.

#### Neuroimaging Correlates of Psychiatric Vulnerability in Prader–Willi Syndrome

Although neuroimaging data in Prader–Willi syndrome remain limited and heterogeneous, emerging findings provide preliminary support for circuit-level consequences of imprinting-related neurodevelopmental disruption. Resting-state functional MRI studies have reported altered functional connectivity within hypothalamic–limbic circuits, as well as dysregulation of default mode and salience networks, which are critically involved in emotional regulation, cognitive control, and psychiatric vulnerability [35,39]. In particular, reduced hypothalamic connectivity has been observed in pediatric cohorts of PWS, with alterations in resting-state functional coupling between the hypothalamus and other cortical regions indicating disrupted network organization in early development [35]. Additionally, neuroimaging studies have demonstrated alterations in frontal reward and limbic circuits, as well as in default mode and salience network connectivity in individuals with PWS, suggesting broader network dysregulation [39,40]. Although sample sizes are small and methodological heterogeneity remains a limitation, recent evidence also indicates structural-functional decoupling of brain networks in children with PWS, further supporting the notion of network instability arising from neurodevelopmental perturbations [41]. Structural MRI findings of altered hypothalamic anatomy in PWS provide complementary support for this multi-level perspective [42].

### 6.3. Integrative Mechanistic Framework: From Imprinting to Behavior

Using information already available, our proposed comprehensive model outlines that defective imprinting can lead to both epigenetic and transcriptional dysregulation, resulting in less than normal differentiation of neurons, abnormal synaptic plasticity, and dysfunctional neurotransmitter systems. We hypothesize these molecular and cellular alterations contribute to network-level circuit instability, leading to neurobehavioral phenotypes including compulsivity, dysregulated emotion, and higher susceptibility to psychosis, based on the type of genotype.

In this conceptualized model, PWS serves as a natural model illustrating the relationship among genomic imprinting, developmentally related psychiatric disorders, and their potential translational relevance as a research model to study how regulatory mechanisms through epigenetics determine brain structure and function and modulate behavior.

**Figure 2:**
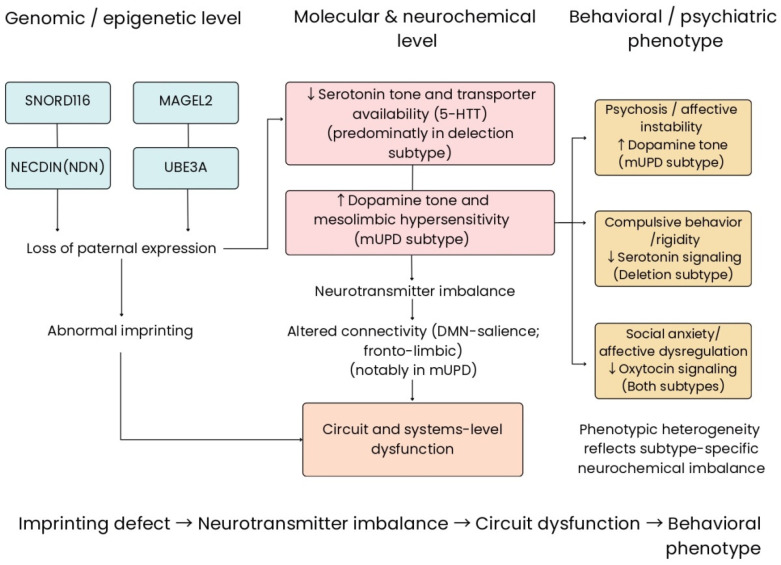
Conceptual, hypothesis-generating framework illustrating proposed multi-level associations between paternal imprinting defects in the chromosome 15q11–q13 region and neurobehavioral phenotypes observed in Prader–Willi syndrome (PWS). The figure integrates genetic and epigenetic alterations (including paternal deletions, maternal uniparental disomy, and imprinting center [PWS-IC] abnormalities) with downstream molecular, neurochemical, and circuit-level findings reported in the literature. Disruption of imprinting-dependent gene expression is depicted as being associated with epigenetic and transcriptional dysregulation, neuromodulatory imbalance, and alterations in large-scale prefrontal–limbic connectivity. The relationships shown are based on convergent but predominantly correlational evidence, particularly from human studies, and are intended to highlight potential pathways linking imprinting abnormalities to neuropsychiatric vulnerability rather than to imply direct causal mechanisms. Solid arrows denote proposed mechanistic associations across biological levels. Genes within the 15q11–q13 region are shown schematically to illustrate imprinting architecture. UBE3A is included for genomic context only, as it is primarily implicated in Angelman syndrome and is not considered a primary pathogenic driver of PWS. This figure represents a hypothesis-generating conceptual model based on correlational evidence rather than direct causal relationships. Figure created by the authors using Canva (Canva Pty Ltd., version current at the time of manuscript preparation); no copyrighted or third-party graphical elements were used.

The conceptual model depicted in Figure 2 illustrates molecular and cellular pathways (chromatin regulation, receptor trafficking, and non-coding RNA), contributing to dysfunctional circuits at the network level. It was developed as a conceptual model and not based upon any specific data.

## 7. Discussion

These studies collectively synthesize multi-level data that may help illustrate how the loss of paternal expression genes on chromosome 15q11–q13 may produce convergent molecular, cellular, and circuit-level disturbances that may influence psychiatric vulnerability in individuals with Prader–Willi Syndrome (PWS). The reviewed studies indicated that regardless of genetic subtype, imprinting errors may trigger a series of events starting with a potential disruption of transcriptional programming and an alteration of the epigenetic state, possibly proceeding through neuromodulatory imbalance and ultimately creating a network-level disorder. Though the mechanisms underlying these disruptions may occur along a common biological axis, there appear to be distinct mechanistic signatures between deletion-type PWS and maternal uniparental disomy (mUPD) that may explain the different neurobehavioral outcomes of these two types of PWS.

At the molecular level, a number of studies have found that the genes *SNORD116*, *MAGEL2*, and *NECDIN* may play important roles in many aspects of neuronal function including neuronal differentiation, synaptic development, intracellular trafficking, and neuromodulatory regulation [10,15,16,17]. Loss of *SNORD116* has been suggested to be a critical component in the post-transcriptional dysregulation of genes in neurons, possibly influencing alternative splicing, chromatin architecture, and networks of genes related to neurogenesis and synaptic formation [17,20]. Similarly to the loss of *SNORD116*, deficiencies in *MAGEL2* and *NECDIN* may disrupt hypothalamic neuropeptide signaling, cytoskeletal organization and stability, axonal stability and proteostasis, thus possibly contributing to the affective rigidity, compulsiveness, and endocrine abnormalities typically seen in individuals with deletion-type PWS [7,21,32].

mUPD, however, results in a much larger-scale disruption of the imprinting architecture. Emerging studies of epigenetics indicate that maternal-only inheritance of chromosome 15q may lead to multilayered silencing through the coordinated deposition of repressive histone modifications and dense methylation at imprinting control regions, resulting in altered chromatin compaction and subsequent transcriptional instability [5,22]. As such, these genome-wide epigenetic disruptions may increase the susceptibility of neurons to dysfunction by disrupting genes outside of the classic PWS locus, specifically those related to neurotransmitter signaling, synaptic maintenance, and circadian circuitry [17,23,24]. This mechanism may provide a basis for the increased risk of mood instability and psychosis-like features observed in mUPD populations [30,34].

Neurochemically, differences between genotypes may be evident in the differential alterations in the serotonergic and dopaminergic systems. Recent studies using knockout mice appear to indicate that *Magel2*-null or *Snord116*-deficient conditions result in serotonergic hyporeactivity [20,27], supporting the hypothesis that decreased serotonergic tone may contribute to compulsive behaviors and emotional rigidity [33]. In contrast, mUPD-associated epigenetic dysregulation may alter the dopamine signaling and reward salience [32,36] that may be responsible for the psychosis-proneness and affective lability [30,34]. Although experimental evidence in humans is currently very limited, the convergence of molecular and neurochemical findings suggests that these mechanisms may be occurring.

Neuroimaging research offers complementary but preliminary evidence for circuit-level involvement. Most studies report altered connectivity in the prefrontal-limbic, default mode, and salience networks, as well as structural anomalies in hypothalamus–pituitary circuitry [30,35]. Due to the variability of studies based on the small sample size, variability of methods, and motion artifacts, it is difficult to draw firm conclusions about the nature of network abnormalities in PWS. However, taken collectively, the data point towards network instability caused by the disrupted neuromodulatory and developmental processes. Additionally, peripheral metabolic and endocrine disturbances, which are common in PWS, could affect these networks through neuroimmune, neurotrophic, or hormonal pathways [37,38].

Overall, the findings presented here provide a rationale for a mechanistic model in which imprinting errors are associated with epigenetic and transcriptional dysregulation, resulting in an abnormality of neuronal maturation and the structure of synapses. It is proposed that the abnormality in neuronal maturation and synaptic architecture will result in an imbalance of neuromodulators, instability of neural circuits, and eventually the emergence of behavioral and psychiatric symptoms. Furthermore, PWS may serve as a model system to explore the relationship between imprinting sensitive pathways and neurodevelopment and psychiatric risk. Additionally, there appear to be similar mechanisms in other disorders that are caused by imprinting errors (e.g., Angelman syndrome) and idiopathic neuropsychiatric disorders (e.g., schizophrenia, affective dysregulation).

Therefore, PWS may serve as a natural experiment to examine how imprinting-sensitive pathways can contribute to neurodevelopment and psychiatric risk. Furthermore, there appear to be similar mechanisms in other disorders that are caused by imprinting errors (e.g., Angelman syndrome) and idiopathic neuropsychiatric disorders (e.g., schizophrenia, affective dysregulation) that may provide a greater degree of translational relevance [31,43].

## 8. Limitations

Despite substantial progress, several limitations may constrain the current mechanistic understanding of PWS. First, many molecular, neurochemical, and imaging studies rely on small cohorts, often combining genetic subtypes or wide developmental ranges, which could reduce statistical power and obscure genotype-specific patterns [30,34,35]. Second, neuroimaging findings remain heterogeneous, likely reflecting variation in acquisition protocols, preprocessing pipelines, and analytic approaches. Third, although animal and cellular models provide high mechanistic resolution, they capture only subsets of the human phenotype and may not fully represent complex gene–environment or developmental interactions. Finally, most evidence linking molecular changes to psychiatric outcomes remains correlational; causal pathways will likely require experimental validation in controlled systems.

## 9. Future Directions

Potential future studies could benefit from stratifying by genotype in a multi-omics approach that includes transcriptomics, methylomics, chromatin topology, and proteomics from either glial or neuronal cells derived from patients. Single-cell analysis would enable researchers to determine which specific cell types are most susceptible to different mutations, whereas brain organoids would allow researchers to study the effects on the development of synapses, as well as the establishment of synaptic networks during the early stages of neurodevelopment in individuals who have an imprinting defect [43]. In addition, the continued advancement of CRISPR technology for modulating epigenetics may enable researchers to develop new tools to experimentally determine whether correcting an imprinting mutation restores cellular, synaptic, and/or neurochemical phenotypic abnormalities (a potential area of investigation at this time) [25]. Researchers may use systems-level analysis by employing multimodal neuroimaging combined with computational modeling to elucidate how molecular disruptions propagate to larger-scale networks [31]. Large longitudinal cohorts of subjects with integrated clinical, behavioral, endocrine/hormonal, and digital phenotyping measures will be necessary to longitudinally assess psychiatric trajectories and identify mechanistic biomarkers associated with increased risk.

## 10. Overall Significance

This review aims to demonstrate how combining evidence from multiple levels of biological organization (genomic, epigenetic, cellular, neurochemical, systems) can help identify Prader–Willi syndrome as a unique disease model in which to investigate how genetic imprinting and epigenetic modification influence psychiatric susceptibility and brain developmental processes. The mechanisms outlined here are likely to be applicable to other neuropsychiatric diseases that arise through similar mechanisms, as well as those caused by rare imprinting syndromes.

## 11. Conclusions

Prader–Willi Syndrome (PWS) is an exceptional and unique disorder to study the influence of imprinting on neurodevelopment, neuromodulation, and psychiatric vulnerability. There is multilevel evidence which indicates that converging impairments in the transcriptional programs, the serotonergic and dopaminergic systems, and the circuits connecting the prefrontal cortex and limbic system, are associated with the distinct behaviorally expressed genotypic phenotypes of PWS. The results of this work will be useful for developing a mechanistic approach to understanding and treating mental illness. They further illustrate the necessity of genotype-stratified biomarker studies, investigation of disorder-specific epigenetic mechanisms, and the development of functional models that recapitulate disease biology to identify therapeutic targets for PWS and related imprinting-associated neuropsychiatric disorders.

## Figures and Tables

**Table 1:** Mechanistic differences between deletion-type and mUPD Prader–Willi syndrome across molecular, neurotransmitter, and circuit levels.

Biological Level	Deletion-Type PWS (15q11–q13 del)	mUPD PWS	Representative References
Primary genetic mechanism	Loss of paternal genes (*MAGEL2*, *SNORD116*, *NDN*)	Maternal uniparental disomy with silencing of paternal allele	Cassidy et al., 2012 [1]; Angulo et al., 2015 [4]
Epigenetic profile	Reduced expression of imprinted genes, mild chromatin dysregulation	Genome-wide imprinting loss; increased methylation; broader chromatin repression	Coulson et al., 2018 [20]; Dong et al., 2024 [26]
Transcriptomic effects	Impaired neuronal maturation, synaptic gene downregulation	Altered expression of neurotransmitter pathway genes; GABA/DOPA shifts	Mercer et al., 2009 [27]; Victor et al., 2021 [28]
Neuromodulatory hypotheses (serotonin/dopamine)	Preclinical evidence suggests serotonergic pathway alterations (e.g., in *Magel2*-null models); direct human neurochemical comparisons cross PWS subtypes remain limited.	Human evidence for subtype-specific monoaminergic profiles is limited; transcriptional/epigenetic studies suggest altered expression of neurotransmitter pathway genes, supporting a hypothesis of neuromodulatory imbalance.	Mercer et al., 2009 [27]
Circuit-level alterations	Hypoactivity in OFC, ACC; impaired top–down control	Prefrontal–limbic dysconnectivity; DMN–salience instability	Whittington & Holland, 2022 [29]; Huang et al., 2023 [30]
Dominant psychiatric phenotype	Compulsivity, affective rigidity, anxiety	Mood instability, psychosis-like symptoms, cognitive-perceptual disturbances	Soni et al., 2007 [31]; Schubert & Schaaf, 2025 [32]
Translational implications	Serotonergic-targeting research models; synaptic maturation target	Dopamine-modulating experimental paradigms; epigenetic therapeutic candidates	Whittington & Holland, 2022 [29]

## Data Availability

No new data were generated or analyzed in this study. All data discussed in the manuscript are derived from previously published sources cited within the article.
